# Supplementary material for: Sustained-input switches for transcription factors and microRNAs are central building blocks of eukaryotic gene circuits
Source: Genome Biol. 2013 Aug 23;14(8):R85. doi: 10.1186/gb-2013-14-8-r85 (PMC4054853; doi:10.1186/gb-2013-14-8-r85)
Supplement: Additional file 5 — HTML Browsable Motif Output. Zipped folder containing all WaRSwap and FANMOD motif output, viewable in a web browser. [file gb-2013-14-8-r85-S5.ZIP › HTML_browsable_motif_output/FANMOD_ath_tair9/sigs_fanmodm-2000.pvals.heatmaps.html/motif_id_38_011011002_tftype_ath_upstream_-1000_0.html]

```
BG_MODEL = FANMOD
MOTIF_ID = 38_011011002
TF_TYPE = ath
UPSTREAM = -1000_0


PVals
FN_0.2	FN_0.4	FN_0.6	FN_0.8
dg_60.genes	0.001	0.042	0.876	0.006
dg_70.genes	0.059	0.021	0.495	0.004
dg_80.genes	0.002	0	0.701	0.205

ZScores
FN_0.2	FN_0.4	FN_0.6	FN_0.8
dg_60.genes	2.873	1.722	-1.187	2.78
dg_70.genes	1.534	2.065	-0.014	2.823
dg_80.genes	2.932	3.85	-0.555	0.763

StDevs
FN_0.2	FN_0.4	FN_0.6	FN_0.8
dg_60.genes	100.411	72.939	33.995	11.644
dg_70.genes	74.267	57.108	26.322	8.943
dg_80.genes	48.893	40.338	19.068	6.559
```
